# Phytophagous Arthropods and a Pathogen Sharing a Host Plant: Evidence for Indirect Plant-Mediated Interactions

**DOI:** 10.1371/journal.pone.0018840

**Published:** 2011-05-18

**Authors:** Raphaëlle Mouttet, Philippe Bearez, Cécile Thomas, Nicolas Desneux

**Affiliations:** French National Institute for Agricultural Research (INRA), UR 880, Sophia-Antipolis, France; Ghent University, Belgium

## Abstract

In ecological systems, indirect interactions between plant pathogens and phytophagous arthropods can arise when infestation by a first attacker alters the common host plant so that although a second attacker could be spatially or temporally separated from the first one, the former could be affected. The induction of plant defense reactions leading to the production of secondary metabolites is thought to have an important role since it involves antagonistic and/or synergistic cross-talks that may determine the outcome of such interactions. We carried out experiments under controlled conditions on young rose plants in order to assess the impact of these indirect interactions on life history traits of three pests: the necrotrophic fungus *Botrytis cinerea* Pers.: Fr. (Helotiales: Sclerotiniaceae), the aphid *Rhodobium porosum* Sanderson (Hemiptera: Aphididae) and the thrips *Frankliniella occidentalis* Pergande (Thysanoptera: Thripidae). Our results indicated (i) a bi-directional negative interaction between *B. cinerea* and *R. porosum*, which is conveyed by decreased aphid growth rate and reduced fungal lesion area, as well as (ii) an indirect negative effect of *B. cinerea* on insect behavior. No indirect effect was observed between thrips and aphids. This research highlights several complex interactions that may be involved in structuring herbivore and plant pathogen communities within natural and managed ecosystems.

## Introduction

Indirect interactions potentially emerge in any community of three or more interacting species [1], [2]. Therefore, complex direct and indirect interactions are expected to occur in ecosystems as species are embedded in large food webs [3]. The occurrence of indirect interactions may represent an important mechanism in determining the establishment and strength of food web interactions in any ecological system [4], [5].

Among indirect interactions, those that are mediated by the first trophic level, i.e. the plant, have received some attention [6], [7] though only a few provisional patterns have been identified. Understanding and identifying these interactions in natural and managed ecosystems is of major importance because plants have to cope with multiple, taxonomically distant second level consumers [8]. Despite obvious differences in damages inflicted to plants by phytophagous arthropods (hereafter named herbivores) and plant pathogens (hereafter named pathogens), potential indirect interactions between these two types of organisms have received limited attention during the last century [7], [9]. For instance, indirect interactions between pathogens and herbivores can occur when infestation by a first attacker changes the shared host plant in a way that affects a second attacker that is often spatially or temporally separated from the first [7]. These interactions potentially impact the life history traits of the attackers, such as herbivore performance [10]–[14] or growth of pathogenic fungi [15]–[20] and thus could be among the leading factors in terms of herbivore and pathogen population dynamics.

While the number of studies on tripartite interactions has increased over past decades [21]–[23], knowledge of the underlying mechanisms is still too limited to be of any predictive value [9]. The most commonly quoted process brings to the forefront finely tuned plant defensive responses following attacks by different enemies [24]. Studies aiming at identifying processes have mainly explored the interactions among signaling cascades, coming up with two broad, non-exclusive cases: synergies and/or antagonisms among signaling pathways [25]. However, the impacts of attacker feeding strategies and spatio-temporal patterns of attacks occurring remain unclear and scarcely documented [7]. Such factors could be of crucial importance in establishing indirect interactions since: (i) the occurrence, the nature (positive, negative or neutral) and the strength of interactions are expected to differ depending on the types of attackers (notably because of various feeding strategies) [26], [27], (ii) the effects are thought to be stronger locally than systemically as the plant may show stronger local responses following an attack [28], [29], (iii) the temporal separation between attacks may determine the state of plant resistance [30], [31].

In this context, our aim was to investigate the indirect interactions among three rose pests and to assess their effect on herbivore and plant pathogen performances. The organisms studied were the plant pathogen *Botrytis cinerea* Pers.: Fr. (Helotiales: Sclerotiniaceae), the aphid *Rhodobium porosum* Sanderson (Hemiptera: Aphididae) and the thrips *Frankliniella occidentalis* Pergande (Thysanoptera: Thripidae). *Botrytis cinerea* has a necrotrophic lifestyle. It kills the host-infected cells and degrades the plant tissue in order to convert it into fungal biomass [32]. *Frankliniella occidentalis* is a cell-content feeding insect. It feeds on the mesophyll and epidermal cells using its mouthparts to lacerate and damage cell tissues [33]. *Rhodobium porosum* is a piercing-sucking (phloem-feeding) insect that uses its stylets to traverse the cuticle, epidermis and mesophyll in order to establish intimate feeding sites in the phloem veins [34]. The study was undergone on a woody plant model; *Rosa hybrida* L. Defense strategy of such plant type differs greatly from herbaceous model plants [35] on which most studies have been carried out. Therefore, our study aimed at increasing current knowledge on indirect plant-mediated interactions.

## Materials and Methods

### Study organisms

The plants used in the experiments were young rose plants, *R. hybrida* cv. Sonia obtained using in vitro synthesis cultivation. They were grown over a nine-week period in climatic chambers (L:D 16∶8, 24±1°C, 65±5% RH). The occurrence of any abiotic or biotic stress was minimized by a daily application of nutrient solution and prophylactic measures to prevent the presence of any pest in the climatic chambers. Plants used for all experiments were of equal size (25±2 cm), used only once, and had never been in contact with plant pathogens or herbivores.

The *B. cinerea* isolate T4 [36] used in this study was provided by Anne-Sophie Walker (INRA Versailles, France). Spores were obtained from mycelium cultivation on malt-agar Petri dishes, after the growing mycelium had covered the entire Petri dish surface. Spore suspensions were prepared by homogenizing the Petri dish content with a Potato Dextrose Broth solution. After centrifugation to remove mycelial debris, spore concentration was measured in a Mallasez® cell and adjusted to a concentration of 10^5^ spores/ml. The herbivores (*R. porosum* and *F. occidentalis*) were reared on caged rose plants (*R. hybrida* cv. Sonia) in climatic chambers (L:D 16∶8, 24±1°C, 65±5% RH).

### Cross-infestation experiments

The influence of pre-infestation by a first attacker on the performance of a second attacker was assessed by realizing cross-infestations on plants. Three-level spatial treatment as well as three-level temporal treatment for attacks were compared.

The spatial design used control plants without any pre-infestation, locally pre-infested plants i.e. pre-infestation on the same leaf but on a different leaflet, and systemically pre-infested plants i.e. pre-infestation on two different leaves from adjacent nodes (mean distance between the two nodes: 4.9±0.3 cm). For systemic pre-infestation, the first attacker was put on the lower leaf and the second on the upper leaf. In most cases, the second attacker was constrained to the terminal leaflet whereas the first attacker was put on a secondary leaflet. In order to constrain the organisms on their respective leaflets and prevent any direct interaction, pathogens and insects were placed inside clip cages composed of a Petri dish cover (4.3 cm diameter) whose outer rim was covered with foam ring, all of which was attached to a fiberglass stake. Insect clip cages had a circular opening made of nylon mesh netting (350 µm) [37], [38]. When the thrips were the second attackers, a microcosm device was preferred to the use of clip cages since reproduction of *F. occidentalis* individuals in clip cages was practically difficult. A microcosm consisted of a plant enclosed in an acryl-glass cylinder (10 cm diameter; 23 cm high) with an upper opening covered by nylon mesh [39], [40]. Hence, no distinction was made between local and systemic pre-infestation in that specific case (only two spatial cases were tested: control and pre-infested plants).

The temporal design refers to the separation between the first and the second infestation by organisms studied. Temporal separations tested were one, four or seven days – respectively called short-term, mid-term or long-term pre-infestation. The first attacker was left on the plant during the complete experiment time-span. Experiments followed an incomplete factorial design (Table 1).

**Table 1:** Spatiotemporal design of cross infestation experiments and replicates undergone per experiment.

Group	Temporal separation	Spatial separation	n
*R. porosum* → *B. cinerea*	Short-term (1 day)	Control / Local / Systemic	34/34/34
	Mid-term (4 days)	Control / Local / Systemic	21/21/21
	Long-term (7 days)	Control / Local / Systemic	25/25/25
*F. occidentalis* → *B. cinerea*	Short-term (1 day)	Control / Local / Systemic	24/24/24
	Long-term (7 days)	Control / Pre-infested	16/16
*B. cinerea* → *R. porosum*	Short-term (1 day)	Control / Local / Systemic	30/30/30
	Mid-term (4 days)	Control / Local / Systemic	14/14/14
	Long-term (7 days)	Control / Local / Systemic	24/18/30
*B. cinerea* → *F. occidentalis*	Short-term (1 day)	Control / Pre-infested	35/35
	Long-term (7 days)	Control / Pre-infested	19/19
*R. porosum* → *F. occidentalis*	Long-term (7 days)	Control / Pre-infested	19/19
*F. occidentalis* → *R. porosum*	Long-term (7 days)	Control / Local / Systemic	24/14/14

Infestations by the pathogen (pre-infestation as first attacker and infestation as second attacker) were realized by dropping 10 µL of spore suspension (10^5^ spores/ml) on the adaxial leaf surface. When used as first attackers, pre-infestation by aphids or thrips was realized by placing 10 individuals on the abaxial leaf surface. When used as second attackers, infestation by aphids consisted in placing three adults on the abaxial leaf surface and infestation by thrips consisted in introducing ten adults inside the microcosm.

Based on pilot experiment results, the relevant performance parameters chosen and recorded for the second attackers were the lesion size of the pathogen (*B. cinerea*) after 4 days, the number of aphids (*R. porosum*) after 4 days and the number of thrips larvae (*F. occidentalis*) after 10 days.

### Behavioral experiments (dual-choice assays)

The influence of fungal pre-infestation on the choice made the herbivores was assessed by providing them with *R. hybrida* leaf discs (diameter: 25 mm) cut out from (i) healthy leaves, (ii) leaflets from leaves previously infested locally by *B. cinerea*, or (iii) leaflets from adjacent leaves previously infested systemically by *B. cinerea* (control, local and systemic groups respectively, hereafter named as such). Herbivores (10 thrips or 10 aphids) were offered two disc types at the same time in a Petri dish (diameter: 5.3 cm) and allowed to settle on, move on and between the different discs for 15 minutes. The number of insects present on each disc was recorded after 15 minutes. The experiment was replicated 27 times with aphids and 26 times with thrips.

### Statistical analyses

A Shapiro–Wilk test was used to check normality of data. For cross infestation experiments, the size of fungal lesion area and the number of aphids were compared within the different spatial distributions (spatial factor) and a generalized linear model (GLM) with a Poisson distribution and a log link function was used. Subsequently, additional GLM analyses followed by Tukey's post hoc tests for multiple comparisons inside temporal sub datasets were carried out. In the specific case of thrips populations for which normalization could not be achieved, the effect of pre-infestation on thrips population was assessed using Wilcoxon rank-sum tests. Finally, data from dual-choice assays were examined with Wilcoxon rank-sum tests (Dunn–Sidak adjustment for multiple comparisons). All analyses were performed with the R statistical software system.

## Results

### Indirect interactions of herbivores on the pathogen

#### Indirect effects of aphids on pathogen

**Figure 1:**
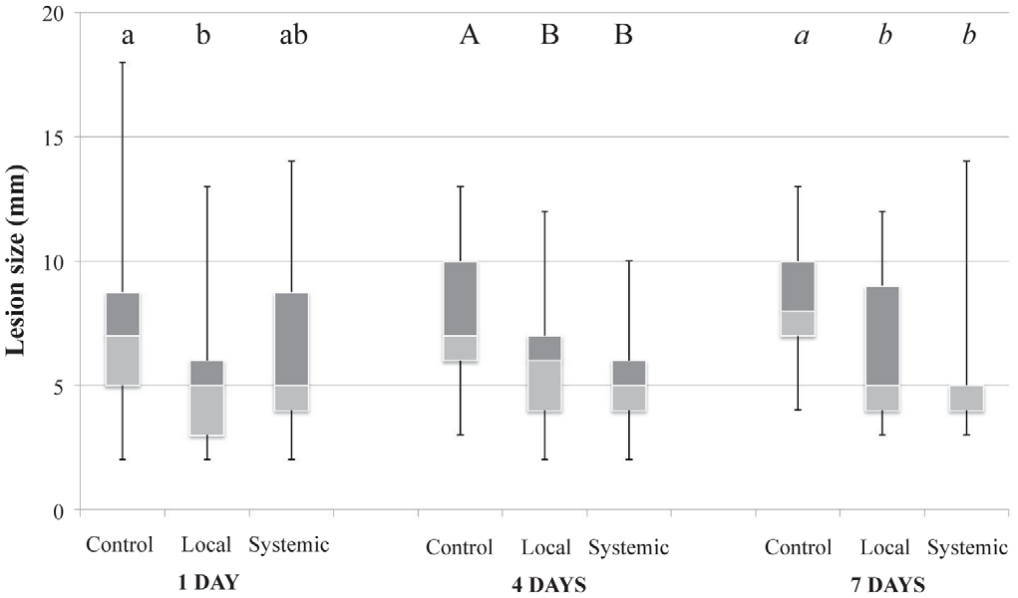
Fungal lesion size following pre-infestations by aphids. Data are presented as smallest observation, lower quartile, median, upper quartile and largest observation. Values for columns bearing different letters within the same temporal delay are significantly different at *P*<0.05.

Fungal lesion size differed according to the spatial disposition of the pre-infestations by aphids (χ^2^ = 30.85, df = 2, *P*<0.001) (Fig. 1). Multiple comparisons within each temporal group showed an indirect negative interaction between *R. porosum* and *B. cinerea*. A short term (1 day) pre-infestation by *R. porosum* induced a 27% decrease in growth of *B. cinerea* lesions (5.35 mm vs. 7.29 mm) in case of local infestation of plants by aphids when compared to control group (χ^2^ = 10.17, df  = 2, *P* = 0.006) (Z = 3.17, *P* = 0.004). No significant difference was observed between control and aphid-systemic infested plants (Z = 1.58, *P* = 0.25), nor between aphid-systemic and aphid-local infested (Z = 1.61, *P* = 0.24). When the pre-infestation by aphids was 4 days, significant reductions in lesion size were observed in both aphid-local (25%) and aphid-systemic (34%) infested plants compared to those observed on control plants (χ^2^ = 12.73, df  = 2, *P* = 0.002) (local: Z = 2.45, *P* = 0.038; systemic: Z = 3.40, *P* = 0.002). The size of *B. cinerea* lesions did not differ between aphid-local and aphid-systemic plants (Z = 0.98, *P* = 0.590). The same trend was observed when the pre-infestation of plants by aphids occurred 7 days before infestation by *B. cinerea* (long-term pre-infestation) with a reduction in size of *B. cinerea* lesions in both aphid-local (26%) and aphid-systemic (36%) infested plants compared to those observed on control plants (χ^2^ = 15.67, df  = 2, *P*<0.001) (local: Z = 2.64, *P* = 0.023; systemic: Z = 3.89, *P*<0.001). No difference in growth of *B. cinerea* lesions was observed between both aphid-pre-infested plants (Z = 1.77, *P* = 0.179).

#### Indirect effects of thrips on pathogen

**Figure 2:**
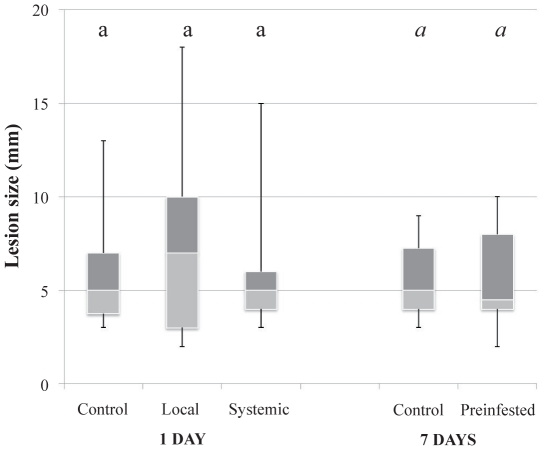
Fungal lesion size following pre-infestations by thrips. Data are presented as smallest observation, lower quartile, median, upper quartile and largest observation. Similar letters indicate no significant differences at *P*<0.05.

No significant difference between the mean size of *B. cinerea* lesions was observed between control plants and those pre-infested by thrips (χ^2^ = 2.86, df  = 3, *P* = 0.415) before infestation by *B. cinerea* (Fig. 2) suggesting that growth of lesion areas of *B. cinerea* was not affected by pre-infestations of the plants by the thrips *F. occidentalis*.

### Indirect interactions of the pathogen on herbivores

#### Indirect effects of pathogen on aphids

**Figure 3:**
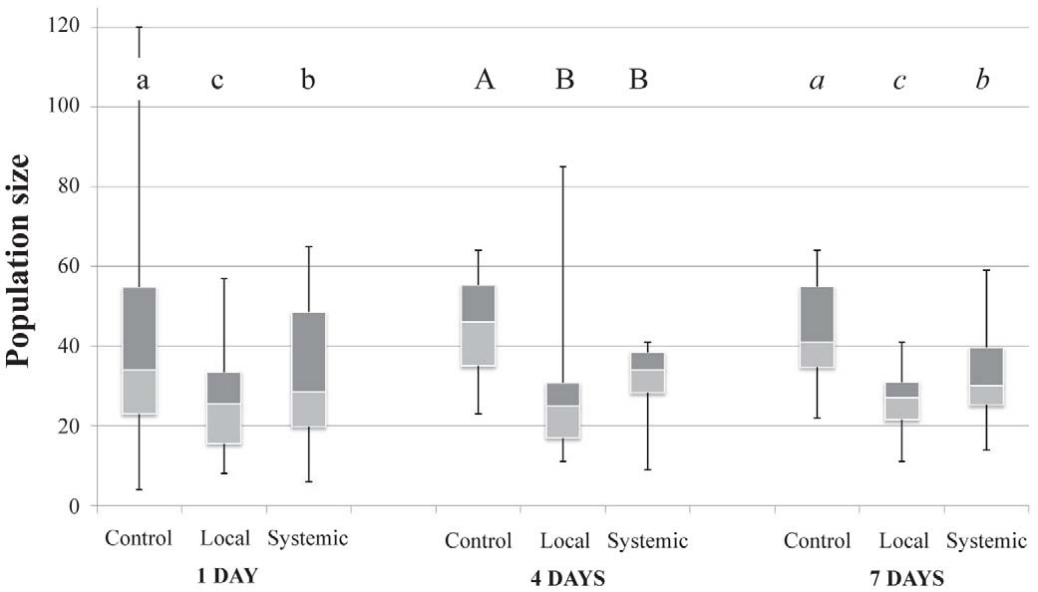
Aphid population size following fungal pre-infestations. Data are presented as smallest observation, lower quartile, median, upper quartile and largest observation. Values for columns bearing different letters within the same temporal delay are significantly different at *P*<0.05.

Aphid population growth was significantly affected by pre-infestation of the host plants by the pathogen *B. cinerea* (χ^2^ = 229.11, df  = 2, *P*<0.001) (Fig. 3). Regardless of the time span between pre-infestation and the second infestation, the mean number of aphids per clip cage was always lower on *B. cinerea* pre-infested plants than on control plants. A short-term pre-infestation (1 day) led to significant reductions of aphid population growth in both locally (32%) and systemically (18%) infested plants when compared to aphid populations on control plants (χ^2^ = 81.36, df  = 2, *P*<0.001) (local: Z = 8.92, *P*<0.001; systemic: Z = 4.11, *P*<0.001). The same trends were observed in the case of mid and long-term pre-infestations by the pathogen (4 days: χ^2^ = 63.87, df  = 2, *P*<0.001, and 7 days: χ^2^ = 89.31, df  = 2, *P*<0.001, respectively) with significant lower final aphid numbers on both locally (mid-term: 38% decrease, Z = 7.54, *P*<0.001; long-term 40% decrease, Z = 8.84, *P*<0.001) and systemically (mid-term: 25% decrease, Z = 5.56, *P*<0.001; long-term: 25% decrease, Z = 6.50 , *P*<0.001) pre-infested plants when compared to control plants.

Regarding the impact of temporal separations between the first and the second infestations, when temporal separation was short i.e. 1 day, the decrease in aphid population growth (lower final aphid numbers) was higher on locally-infested plants than on systemically-infested ones (Z = 4.89, *P*<0.001). With a mid-term separation, no significant difference was observed between both positions (Z = 2.01, *P* = 0.097) although a higher reduction in aphid growth (marginally significant) was clearly observed on the local position than on the systemic one (Fig. 3). As for the short-term, a long-term separation led to a higher decrease in populations of locally-infested plants compared to the systemically-infested ones (Z = 3.55, *P* = 0.001).

**Figure 4:**
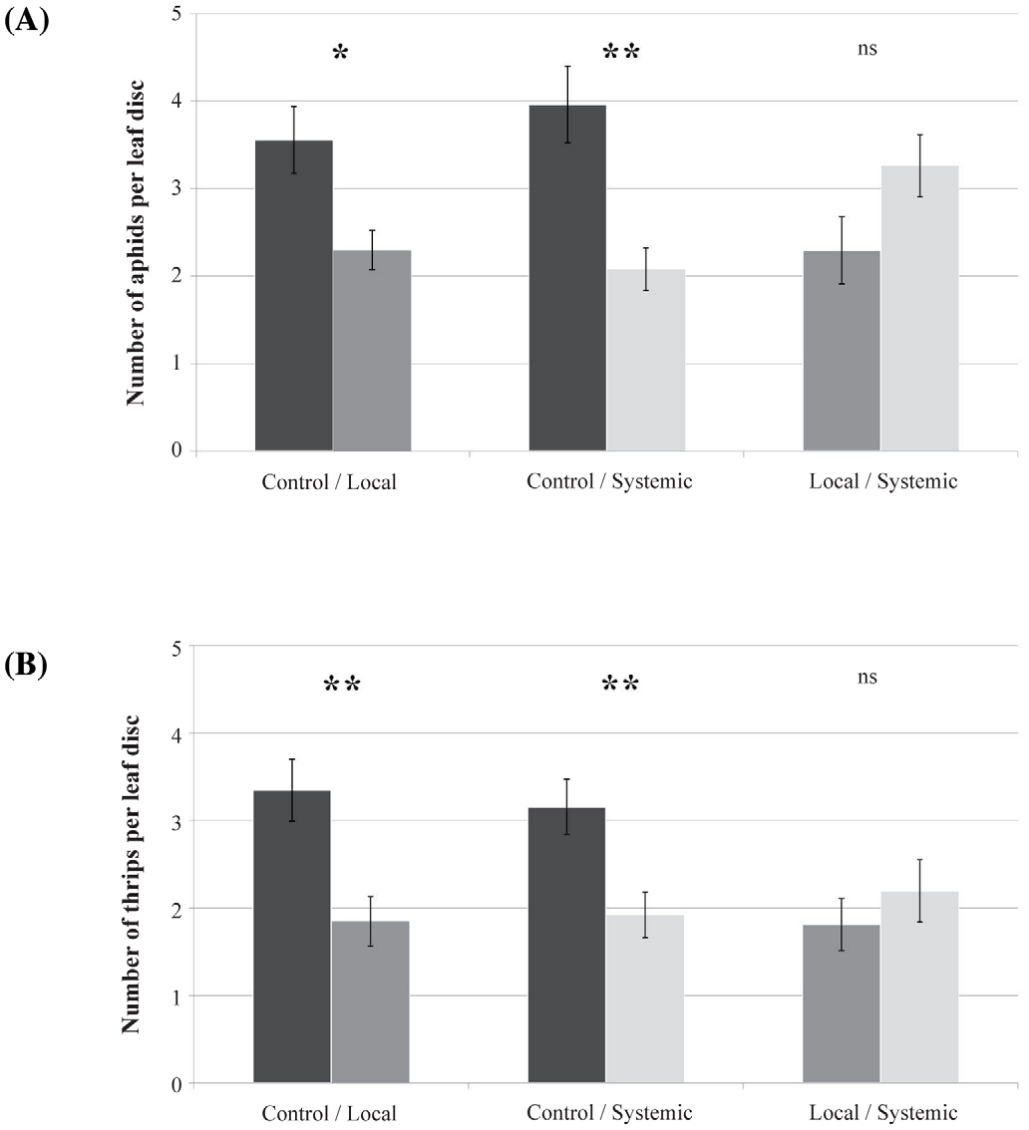
Distribution of phytophagous arthropods on leaf discs in dual choice assays after 15 minutes. (A) Mean (± SEM) numbers of aphids. (B) Mean (± SEM) numbers of thrips. Asterisks denote significant Wilcoxon tests comparing treatments (*: *P*<0.05, **: *P*<0.01, ns: not significant).

During the dual-choice assay, *R. porosum* individuals showed a marked preference for leaf discs from control plants over those from *B. cinerea*-infested plants (Fig. 4A). In the choice tests between control and locally or systemically-infested disc leaves, the mean number of aphids settling on control leaf discs was significantly higher than those on the locally-infested leaf discs (W = 505, *P* = 0.014) and than those on the systemically-infested leaf discs (W = 547, *P* = 0.001). By contrast, no significant difference was observed between systemically- and locally-infested leaf discs (W = 261, *P* = 0.071) though there was a marginally significant trend for aphids to prefer the systemically to locally-infested leaf discs.

#### Indirect effects of pathogen on thrips

**Figure 5:**
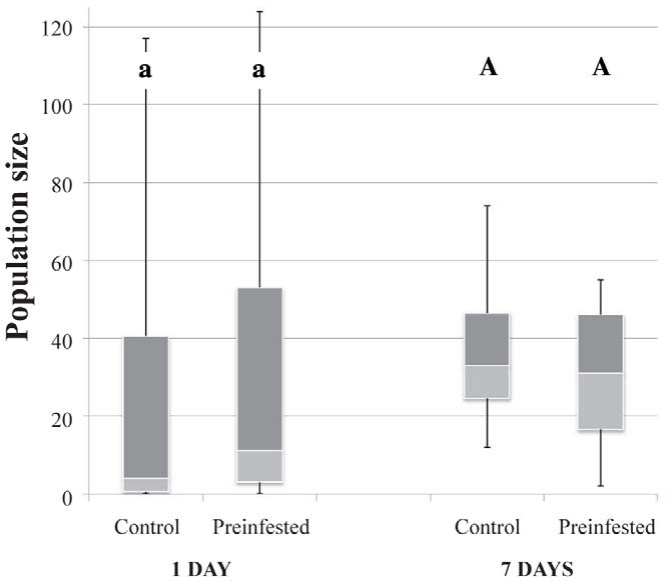
Thrips population size following fungal pre-infestations. Data are presented as smallest observation, lower quartile, median, upper quartile and largest observation. Similar letters indicate no significant differences at *P*<0.05.

The thrips population growth was not significantly affected by the short-term pre-infestation of plants with the pathogen *B. cinerea* (W = 487.85, *P* = 0.142), nor was it affected by a long-term pre-infestation (W = 204, *P* = 0.502) (Fig. 5).

During the dual-choice assay, *F. occidentalis* individuals showed a marked preference for leaf discs from control plants over those from *B. cinerea*-infested plants (Fig. 4B). In the choice test between control and locally-infested leaf discs, the mean number of thrips settling on control discs was significantly higher than on infested discs (W = 500.5, *P* = 0.002). The same trend was observed when comparing the number of thrips choosing control discs over systematically-infested leaf discs (W = 489, *P* = 0.005). No difference was observed between leaf discs from locally and systemic-infested plants (W = 376, *P* = 0.485).

### Indirect interactions between the two herbivores

**Figure 6:**
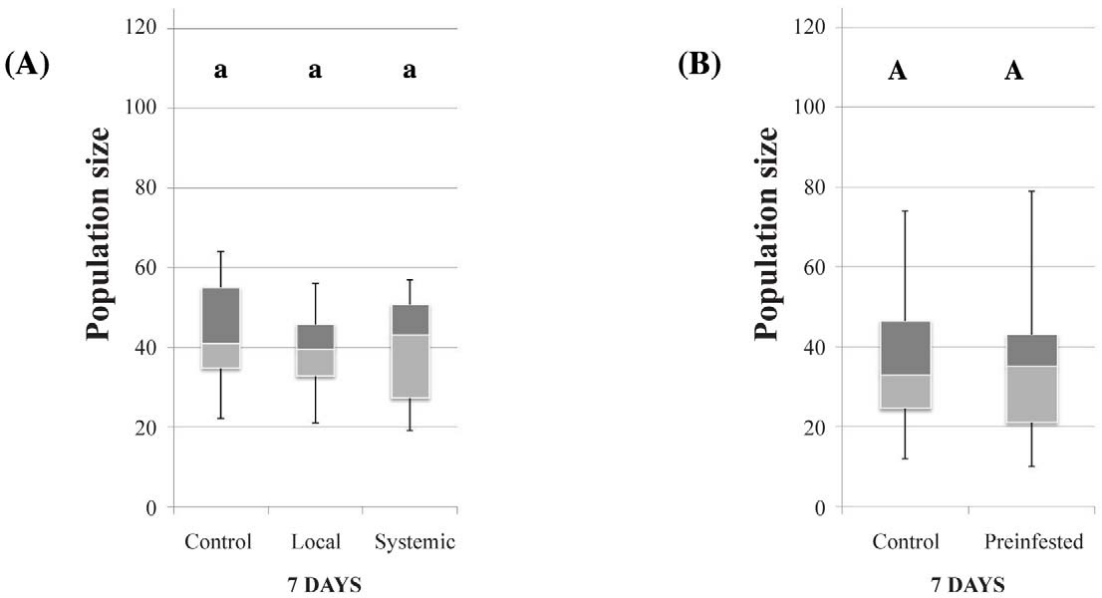
Aphid (A) and thrips (B) population size following pre-infestation of plants with thrips or aphids respectively. Data are presented as smallest observation, lower quartile, median, upper quartile and largest observation. Similar letters indicate no significant differences at *P*<0.05.

Neither aphid (Fig. 6A) nor thrips (Fig. 6B) population growth was affected by the pre-infestation of plants with thrips and aphids respectively (χ^2^ = 4.75, df  = 2, *P* = 0.093 and W = 185.5, *P* = 0.895).

## Discussion

The present study enabled us to identify indirect interaction patterns between a plant pathogen and herbivorous arthropods. A bi-directional detrimental effect on performance was observed between *B. cinerea* and *R. porosum*, conveyed by decreased aphid growth rate and a decreased fungal lesion area, whereas no effect on performance was observed between *B. cinerea* and *F. occidentalis* or between *F. occidentalis* and *R. porosum*.

These results demonstrate that occurrence of plant-mediated indirect interactions between species may depend on their feeding strategies on the plant. Regarding potential mechanisms involved, *B. cinerea* and *R. porosum* may induce pathways that share similarities in such a way that the first attack enhances the plant's ability to resist the second attacker. Plant responses to piercing-sucking insects overlap responses activated against plant pathogens [26], [41] and aphids have been proven to activate the salicylic acid (SA)-dependent pathway as well as triggering off plant resistance (R) genes [42], [43]. By contrast, when *F. occidentalis* was involved in cross-infestation experiments, indirect interactions were not observed. Cell-content feeder insects, e.g. thrips, are thought to induce the jasmonic acid (JA)-regulated wound-response pathway and its associated octodecanoid pathway [44], [45]. Different signaling pathways could regulate varied plant responses that are effective against distinct types of attackers [46]. Regarding the two major pathways, i.e. the SA-dependent and the JA-dependent pathways, indirect negative interactions may arise between attackers that induce similar signaling pathways [47]. On the other hand, indirect positive interactions may occur between two attackers when one of them induces one pathway and the other one another [48], [49]. This can arise because of the well-documented antagonistic relationship between the JA and SA pathways [50]–[52]. Another process that may explain the occurrence of indirect interactions between attackers is the production of secondary metabolites following the first attack. To our knowledge research on such metabolites have not been undertaken on roses, although pilot experiments carried out in our laboratory (High Performance Liquid Chromatography analysis of leaflets from cross-infestations experiments) suggest that *R. hybrida* does respond to attacks by diverse enemies using various defense responses (Mouttet R., Ponchet M. and Desneux N., unpublished data). It is possible that infestation by *B. cinerea* leads to a production of a relatively broad spectrum of defense metabolites that affect the performance of *R. porosum*, and vice versa. By contrast, *F. occidentalis* would not be affected by these metabolites, nor would *B. cinerea* and *R. porosum* be affected by secondary metabolites produced following a first attack by *F. occidentalis*. Since plants could have quantitative metabolites targeting a wide range of pathogens as well as herbivores [53]–[55], it would be relevant to study the multiple secondary metabolites produced by plants. All the more so, *B. cinerea* was shown to affect the behavior of both *R. porosum* and *F. occidentalis*: in a choice situation, the insect preferred to feed on healthy leaflets than on infested ones. This observed preference could be explained by the production of secondary metabolites that have a toxic, antifeedant or aversive effects on insects after *B. cinerea* plant infection.

Our results shed light on the importance of the spatio-temporal design of experiments in shaping the indirect interactions between *B. cinerea* and *R. porosum*. Detrimental effects on performance were observed at the local level in every case. It was at least similar if not stronger than the potential systemic effect. Indeed, the most drastic changes in the plant might occur at the local level to restrain the infection or infestation locally [9], [56]. A weaker systemic response could allow the plant to face further attacks while limiting the cost of defense responses [57], [58]. Indeed, the Optimal Defense Theory states that, under high risk of attack, uneven distribution of defense components confers enhanced protection of plant tissues without overly compromising plant fitness [59]. The temporal aspect that determines the state of plant resistance may also be considered within the context of the overall defense strategy of the host plant, which requires the allocation of resources away from growth and reproduction [35]. In our study, the indirect interaction between *B. cinerea* and *R. porosum* depended on the time span between infestations. The detrimental effect on the aphid population growth was first conveyed locally by a short-term pre-infestation, and subsequently both locally and systemically by mid and long-term pre-infestations. Regarding the negative effect on the pathogen, it was stronger on the local level with short and long-term pre-infestations whereas no difference between the local and the systemic effects was observed with a mid-term pre-infestation. These patterns suggest that plant defenses can change in space and time following multiple attacks. It has been put forward that “fine tuning” resistance to biotic threats in plants lies in the spatio-temporal patterns of its induction [60]. A time span delay in the display of defense mechanisms could offset its fitness cost by optimizing the allocation of resources while ensuring the plant copes efficiently with multiple attackers [27]. Understanding such complex mechanisms would require further studies, including a biochemical approach, in order to investigate, characterize and monitor the plant defense responses that mediate these indirect interactions in time and space [24], [61], [62].

Last but not least, plant-mediated indirect interactions can impact plant pathogen and herbivore population dynamics. For instance, we observed a decreased pathogen growth up to 36% following pre-infestation by aphids, and a decreased aphid population growth up to 40% following pathogen pre-infestation. This confirms the role of indirect interactions in molding the structure of species assemblages [63]. Nevertheless, it is essential to transfer the results obtained under controlled conditions to larger and more realistic scales in which environmental conditions are less controlled and many direct and/or indirect biological interactions may take place. Moreover, in a given environment, the presence of various plant phenotypes (i.e. infested or non-infested by pathogens) and the ability of herbivores to discriminate between healthy and infested plants could influence the spatial distribution of herbivorous arthropods [64].

Applied perspectives promoting negative indirect interactions such as the effect of *R. porosum* on the performance of *B. cinerea* could be useful for Integrated Pest Management (IPM) purposes and lead to reduced use of pesticides which are noxious to beneficial arthropods [65]. It could be true for greenhouse rose crops in particular where fungicides are extensively used against plant pathogens [66]. This is of great concern since a sound understanding of ecological processes that drive population dynamics is a pre-requisite for the design of adequate IPM strategies [67]–[70].

This study provides valuable insight into how diverse attackers with different feeding strategies can interact indirectly, depending on the spatio-temporal heterogeneity of biotic attacks. However, only through a parallel biochemical approach will it be possible to elucidate the complex mechanisms that mold indirect plant-mediated interactions. In addition, further research is needed to address the relative importance of these interactions in structuring herbivore and plant pathogen communities in natural growing conditions.
